# Robust neuroinflammation and perivascular pathology in rTg-DI rats, a novel model of microvascular cerebral amyloid angiopathy

**DOI:** 10.1186/s12974-020-01755-y

**Published:** 2020-03-04

**Authors:** Xiaoyue Zhu, Joshua Hatfield, Joseph K. Sullivan, Feng Xu, William E. Van Nostrand

**Affiliations:** 1 Department of Biomedical and Pharmaceutical Sciences, College of Pharmacy, George & Anne Ryan Institute for Neuroscience, University of Rhode Island, 130 Flagg Road, Kingston, RI 02881 USA; 2 Present Address: Cold Spring Harbor Laboratory, 1 Bungtown Road, Cold Spring Harbor, NY 11724 USA; 3 Present Address: New York Medical College, 40 Sunshine Cottage Road, Valhalla, NY 10595 USA

**Keywords:** Cerebral amyloid angiopathy, Alzheimer’s disease, Transgenic rats, Beta amyloid, Neuroinflammation, Perivascular stress

## Abstract

**Background:**

Cerebral amyloid angiopathy (CAA) is a common cerebral small vessel disease of the aged and a prominent comorbidity of Alzheimer’s disease (AD). CAA can promote a variety of vascular-related pathologies including neuroinflammation, cerebral infarction, and hemorrhages, which can all contribute to vascular cognitive impairment and dementia (VCID). Our understanding of the pathogenesis of CAA remains limited and further investigation of this condition requires better preclinical animal models that more accurately reflect the human disease. Recently, we generated a novel transgenic rat model for CAA (rTg-DI) that develops robust and progressive microvascular CAA, consistent microhemorrhages and behavioral deficits.

**Methods:**

In the current study, we investigated perivascular pathological processes that accompany the onset and progressive accumulation of microvascular CAA in this model. Cohorts of rTg-DI rats were aged to 3 months with the onset of CAA and to 12 months with advanced stage disease and then quantitatively analyzed for progression of CAA, perivascular glial activation, inflammatory markers, and perivascular stress.

**Results:**

The rTg-DI rats developed early-onset and robust accumulation of microvascular amyloid. As the disease progressed, rTg-DI rats exhibited increased numbers of astrocytes and activated microglia which were accompanied by expression of a distinct subset of inflammatory markers, perivascular pericyte degeneration, astrocytic caspase 3 activation, and disruption of neuronal axonal integrity.

**Conclusions:**

Taken together, these results demonstrate that rTg-DI rats faithfully mimic numerous aspects of human microvascular CAA and provide new experimental insight into the pathogenesis of neuroinflammation and perivascular stress associated with the onset and progression of this condition, suggesting new potential therapeutic targets for this condition. The rTg-DI rats provide an improved preclinical platform for developing new biomarkers and testing therapeutic strategies for microvascular CAA.

## Background

Cerebral amyloid angiopathy (CAA) is a prominent cerebral small vessel disease characterized by the deposition of fibrillar amyloid beta peptide (Aβ) within small arteries and arterioles of meninges and cortex as well as the brain capillaries [1, 2]. Sporadic CAA is common in the elderly brain and is present in > 50% of individuals over the age of 80 years [3]. It has been reported that greater than 80% Alzheimer’s disease (AD) patients present CAA pathology in varying levels [1–4]. Further, early-onset, familial forms of CAA result from specific mutations within the Aβ peptide including the Dutch E22Q and Iowa D23N variants [5–7]. Clinically, CAA can promote cerebral infarction, intracerebral hemorrhages (ICH), and microbleeds [1, 2, 8, 9]; all of which can contribute to vascular cognitive impairment and dementia (VCID).

There are two prominent forms of CAA. When restricted to the meningeal and intracortical cerebral arterioles, this is referred as CAA type-2 [1, 10]. In CAA type-1, the deposition of Aβ is present on the capillary walls and observed in approximately half of AD cases [10]. In contrast to larger-vessel CAA type-2, CAA type-1 results in fibrillar amyloid penetrating the surrounding brain parenchyma, referred to as dyshorric amyloid, which further promotes a strong perivascular neuroinflammatory response [1, 10–12]. It is suggested that the early pericapillary Aβ impairs the perivascular drainage pathway, which leads to the disruption of Aβ clearance resulting in more Aβ deposition on capillary walls [1]. Further, CAA type-1 is often correlated with impaired cognition and rapidly progressive dementia [13–15]. Although CAA type-1 shows high clinical relevance, its contributions to neurodegenerative diseases is still unclear. Therefore, a better understanding of the mechanisms involved in CAA pathogenesis may be helpful in the design of therapeutic approaches targeting this condition.

Recently, we generated a novel transgenic rat model (rTg-DI) that produces low levels of human familial CAA Dutch/Iowa E22Q/D23N mutant Aβ in the brain [16]. The rTg-DI rat model shows many pathologic aspects of human small vessel CAA type-1, including similar vascular Aβ structure, glial activation, and microhemorrhages, accompanied by behavioral deficits [16]. In order to gain a better understanding of the mechanisms underlying CAA pathology, we investigated perivascular pathological processes that accompany CAA type-1 in the rTg-DI rat model. Here we show that rTg-DI rats exhibit early-onset and progressive accumulation of capillary fibrillar Aβ paralleled with increasing numbers of perivascular glial cells. Furthermore, accumulating vascular amyloid promoted expression of a subset of inflammatory markers, which was accompanied by loss of perivascular pericytes, induction of cellular caspase 3, and axonal pathology. Collectively, this work demonstrates that the rTg-DI model faithfully recapitulates multiple aspects of human CAA type-1 and provides further insight into emerging CAA type-1 pathologies, which may offer new targets in developing appropriate therapeutic interventions.

## Methods

### Animals

The generation of rTg-DI transgenic rats was recently described [16]. These rats modestly express human Swedish/Dutch/Iowa mutant AβPP under the control of the neuronal-specific Thy1.2 promoter. The deposition of vascular fibrillar mutant Dutch/Iowa Aβ peptide begins at around 3 months of age with a progressive increase in the amounts of primarily soluble and insoluble pools Aβ40 in the brain with age. Heterozygous rTg-DI rats and non-transgenic, wild-type rats at 3 and 12 months of age were used in the present study. All work with animals was in accordance with the United States Public Health Service’s Policy on Humane Care and Use of Laboratory Animals and was approved by the University of Rhode Island Institutional Animal Care and Use Committee (IACUC).

### Brain tissue preparation

Animals were euthanized with CO_2_ at specified ages. The rat brain was surgically removed and bisected in the mid-sagittal plane. One hemisphere was snap-frozen in liquid nitrogen for mRNA and protein analyses. The other hemisphere was either fixed in 4% paraformaldehyde or fixed in 70% ethanol followed by xylene treatment and embedding in paraffin or snap-frozen in optimal cutting temperature medium (OCT 4585, Fisher Healthcare) directly.

### Immunohistochemistry

For glial cell analysis, paraffin-embedded brains were sagittally sectioned at 10 μm, or for stereological analysis at 50 μm, thickness using a microtome, deparaffinated and rehydrated. Then sections were incubated with proteinase K (0.2 mg mL^−1^) for 5 min at room temperature. Sections were then blocked in Superblock blocking buffer (37518, ThermoFisher) containing 0.3% Triton X-100 at room temperature for 30 min and incubated with individual primary antibodies at the following dilutions overnight: rabbit polyclonal antibody to collagen IV (1:250, SD2365885, Invitrogen), goat polyclonal antibodies to glial fibrillary acidic protein (GFAP, 1:250, ab53554, Abcam), or ionized calcium-binding adapter molecule 1 (Iba-1, 1:250, NB100–1028, Novus). Mouse monoclonal antibody to OX6 MHCII (1:200, Abcam) was used to identify cells in the brain that potentially represent macrophagic microglia.

For the pericyte staining, the fresh-frozen OCT directly embedded tissues were used. Sections were fixed in acetone for 10 min, then air dried for 30 min followed by PBS rehydration for 5 min at room temperature. Sections were blocked in Superblock blocking buffer for 30 min then incubated with goat polyclonal antibody to platelet-derived growth factor receptor beta (PDGFRβ, 1:250, gov0415021, R&D) and rabbit polyclonal antibody to collagen IV overnight.

For fluorescence staining, after the overnight incubation, sections were washed with PBS for three times, then incubated with Alexa Fluorescent 594- or 488-conjugated secondary antibodies (1:1000). Fluorescent staining for fibrillar amyloid was performed using either thioflavin S (123H0598, Sigma-Aldrich) or Amylo-Glo (TR-300-AG, Biosensis Inc.), as described by the manufacturer. Nuclear staining was performed with 4′,6-diamidino-2-phenylindole (DAPI, 10236276001, Sigma-Aldrich).

### Quantitative measures of CAA pathologies

The percent area of capillaries covered with amyloid was quantified using a set of sections that were stained with thioflavin S and immunolabeled with antibody to collagen IV. A series of non-overlapping images covering the cortex, hippocampus, and thalamus were captured and analyzed by the Image J software. For each field, the sum area of thioflavin S was divided by that for the total collagen IV immunoreactive capillary area × 100 to yield the percent area of microvascular amyloid.

The numbers of astrocytes and microglia, and pericytes in capillaries of the cortex, hippocampus, and thalamus at each 3 and 12 months of age were determined using stereological principles [17]. The density of microglia and astrocytes was quantified in the regions of the cortex, thalamus, and hippocampus of rTg-DI and age-matched WT rats. The total numbers of microglia and astrocytes were estimated using the Stereologer software system (Systems Planning and Analysis). Every tenth section cut at 50 μm was selected and generated 10–15 sections per reference space in a systematic-random manner. Immunopositive cells were counted using the optical fractionator method with the dissector principle and unbiased counting rules [17]. Criteria for counting cells required that cell bodies exhibited positive GFAP or Iba-1 immunostaining, for astrocytes or microglia respectively.

The density of pericytes was calculated using a set of brain sections labeled with antibodies to PDGFRβ for pericytes and collagen IV to identify capillaries/microvessels and Amylo-Glo to reveal deposited fibrillar amyloid. The number of pericyte cell bodies and the length of capillaries were calculated in the BZ-X Analyzer software. Then, the pericyte coverage in each area was calculated by the total number of pericytes divided the sum length of capillaries.

### Real-time quantitative PCR

Rat brains at each age were collected and snap-frozen in liquid nitrogen. The brains were first homogenized within Trizol (162711, Invitrogen), then the total RNA was extracted by using the Direct-zol RNA MiniPrep kit (ZRC200796, ZYMO research) according to the manufacturer’s protocol. Total RNA concentrations were measured using Nanodrop (Nanodrop one, Thermo Scientific). cDNA was synthesized with the High Capacity cDNA Reverse Transcription Kit (00289994, Applied Biosystems) following manufacturer’s guidelines. Quantitative PCR (qPCR) were performed in the Step One Plus Real-time PCR system (Applied Biosystems) using the Taqman primers (CD86, Rn00571654_m1; CD68, TREM2, Rn01512170_m1; Rn01495634_g1; GFAP, Rn00566603_m1; TGFβ1, Rn00572010_m1; TNFα, Rn01525859_g1; IL1β, Rn00580432_m1; IL10, Rn01483988_g1; IL6, Rn01410330_m1; IL17, Rn01757168_m1; C3, Rn00566466_m1; C1q,Rn00595250_m1; C4b, Rn01774112_mH; C1inh, Rn01485600_m1; MMP9, Rn00579162_m1; MMP2, Rn01538170_m1; Actb, Rn00667869_m1). Obtained mRNA expression levels were normalized to Actin-beta.

### Identification of pro-apoptotic cells

The numbers of pro-apoptotic cells were determined using stereological principles as described above. Sections of 3-month and 12-month wild-type and rTg-DI rat brains were immunolabeled with a rabbit antibody for active-caspase 3 for identifying the pro-apoptotic cells (1:250, AF835, Novus), mouse monoclonal antibody 66. 1[18] (1:250) to label fibrillar amyloid and stained with DAPI for nuclear labeling for counting the total number of cells. The ratio of caspase 3-positive cells to total cell number in cortex, hippocampus, and thalamus were calculated using the BZ-X Analyzer software. To identify the caspase 3-positive cell types, the brain sections were double immunolabeled with antibody to caspase 3 and with antibodies to NeuN, GFAP, or Iba-1 to identify neurons, astrocytes, or microglia, respectively.

### Analysis of axonal pathology

Axonal integrity was evaluated by immunolabeling the brain sections from 3 and 12-month-old wild-type and rTg-DI rats with SMI312 mouse monoclonal pan axonal neurofilament marker (1:200, 837904, Biolegend). Tissue sections were stained with thioflavin S to identify microvascular fibrillar amyloid deposits.

### Statistical analysis

Statistical analysis was performed using the Graphpad Prism software. Results are shown as mean value with standard deviation (SD) of the mean. The statistical differences between pairs of data sets were analyzed by *t* test at the 0.05 significance level.

## Results

### Progressive accumulation of microvascular amyloid is accompanied by increased numbers and activation of glial cells

**Fig. 1:**
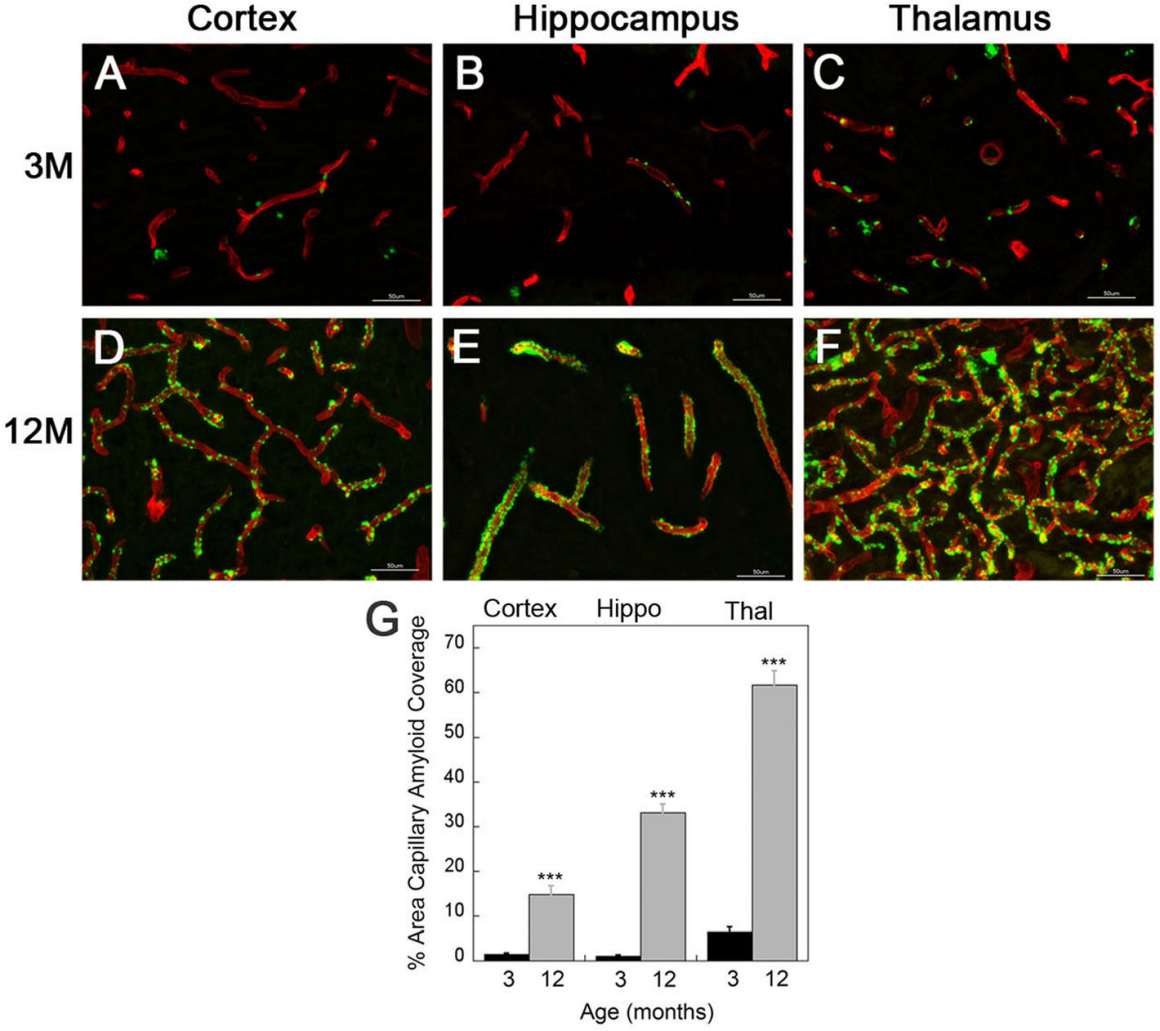
Quantitative analysis of progressive accumulation of microvascular CAA in rTg-DI rats. **a**–**f** Brain sections from 3-month-old (**a**–**c**) and 12-month-old (**d**–**f**) rTg-DI rats were immunolabeled with rabbit polyclonal antibody to collagen IV to specifically detect cerebral microvessels (red) and the thioflavin S to identify fibrillar amyloid (green). The rTg-DI rats showed progressive cerebral microvascular fibrillar amyloid deposition in the cortical (**a**, **d**), hippocampal (**b**, **e**), and thalamic regions (**c**, **f**). Scale bars = 50 μm. **g** Quantitation of cerebral microvascular amyloid load in different brain regions of 3-month-old (black bars) and 12-month-old (gray bars) rTg-DI rats. Data are expressed as means ± SD of *n* = 6–7 rTg-DI rats per group. ***P < 0.001

rTg-DI rats exhibit progressive accumulation of cerebral microvascular fibrillar amyloid in the cortex, hippocampus, and thalamus (Fig. 1). The deposition of microvascular amyloid begins at ≈ 3 months of age in all three brain regions (Fig. 1a–c). At 12 months of age, the rTg-DI rats developed extensive cerebral microvascular Aβ deposition, with more than 30% and 60% of the capillaries covered by fibrillar Aβ in the hippocampus and thalamus, respectively (Fig. 1g). Although the level of CAA was somewhat lower in the cortical area, still more than 15% of the vessel surface was covered with amyloid. These findings demonstrate that the rTg-DI rat model consistently develops early-onset and extensive cerebral vascular Aβ accumulation in the brain. For all of the proceeding analyses, measures were performed at the early-stage onset of microvascular CAA (3 months) and compared with late-stage disease with extensive microvascular CAA (12 months).

**Fig. 2:**
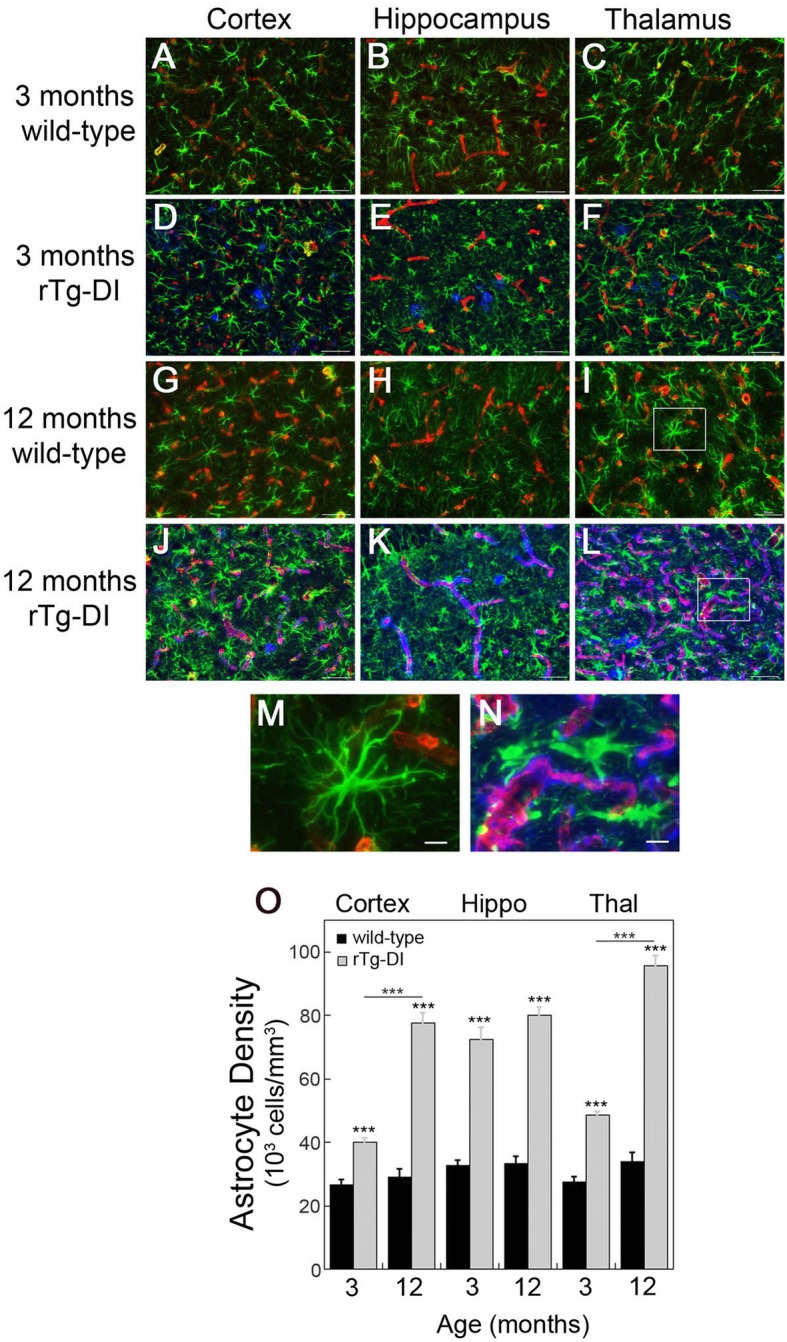
Increased perivascular astrocytes in rTg-DI rats. **a**–**l** Brain sections from 3-month-old wild-type (**a**–**c**) and rTg-DI (**d**–**f**) rats and 12-month wild-type (**g**–**i**) and rTg-DI (**j**–**l**) rats were labeled with Amylo-Glo to detect fibrillar amyloid (blue), rabbit polyclonal antibody to collagen IV to detect cerebral microvessels (red), and goat polyclonal antibody to GFAP to identify astrocytes (green). Scale bars = 50 μm. **m**, **n** Enlarged images of the highlighted regions of panels **i** and **l**, respectively. Scale bars = 10 μm. **o** Quantitation of astrocyte numbers from wild-type rats (black bars) and rTg-DI rats (gray bars) in different brain regions at 3 and 12 months of age. Data shown are mean ± SD of *n* = 5–6 rats per group. Compared to wild-type rats the astrocyte numbers were markedly elevated in rTg-DI rats and increased from 3 to 12 months of age in measured brain regions. ***P < 0.001

Astrocytes have been increasingly recognized as an important contributor to the neuroinflammatory and neurodegenerative processes in Aβ-related human disorders including CAA [19]. The accumulation of cerebral microvascular amyloid in rTg-DI rats similarly promotes a strong astroglial response in several ways. For example, at 3 months of age with the onset of microvascular amyloid deposition, there is a noticeable uptick in the numbers of astrocytes and the beginning of a change in appearance compared to age-matched wild-type rats (Fig. 2a–f, o). These early changes observed in 3 months old rTg-DI rats were not evident in younger 1 month old animals, prior to onset of microvascular amyloid deposition (Additional Fig. 1). As rTg-DI rats aged to 12 months and presented with extensive microvascular CAA (Fig. 1), there was a dramatic increase in the numbers of astrocytes in the cortical, hippocampal, and thalamic regions (Fig. 2 j–l, o) whereas the numbers of astrocytes in these brain regions of wild-type rats remained fairly constant compared to 3 months of age (Fig. 2g–i, o). Further, a pronounced morphological alteration of astrocytes was observed in rTg-DI rats. In contrast to the astrocytes present in the brains of 12-month wild-type rats (Fig. 2m), the astrocytes surrounding microvessels with amyloid generally showed increased cell body volume and thickened, retracted processes (Fig. 2n).

**Fig. 3:**
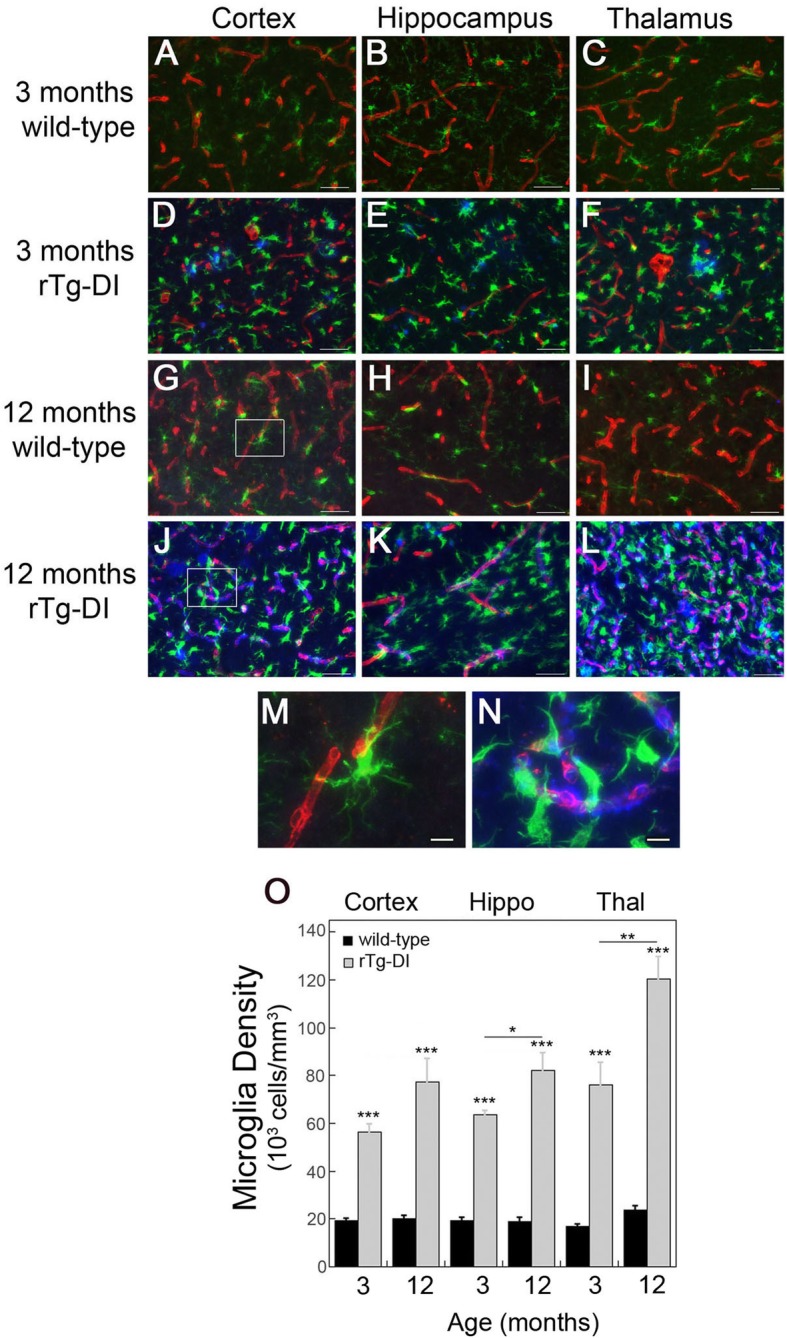
Increased perivascular microglia in rTg-DI rats. **a**–**l** Brain sections from 3-month-old wild-type (**a**–**c**) and rTg-DI (**d**–**f**) rats and 12-month wild-type (**g**–**i**) and rTg-DI (**j**–**l**) rats were labeled with Amylo-Glo to detect fibrillar amyloid (blue), rabbit polyclonal antibody to collagen IV to detect cerebral microvessels (red), and goat polyclonal antibody to Iba-1 to identify microglia (green). Scale bars = 50 μm. **m**, **n** Enlarged images of the highlighted regions of panels **g** and **j**, respectively. Scale bars = 10 μm. **o** Quantitation of microglia numbers from wild-type rats (black bars) and rTg-DI rats (gray bars) in different brain regions at 3 and 12 months of age. Data shown are mean ± SD of *n* = 5–6 rats per group. Compared to wild-type rats the microglia numbers were markedly elevated in rTg-DI rats and increased from 3 to 12 months of age in the measured brain regions. *P < 0.02, **P < 0.01, ***P < 0.001

Activated microglia have also been strongly associated with human CAA type-1 vascular amyloid since it engages the surrounding brain parenchyma [12, 20, 21]. We found that in rTg-DI rats the presence of microvascular amyloid dramatically changes the numbers and morphological state of microglia. At 3 months of age, with onset of microvascular amyloid, the numbers of microglia sharply rose compared to wild-type rats (Fig. 3a–f, o). As the rTg-DI rats further aged to 12 months, presenting with more extensive microvascular amyloid, the numbers of microglia further increased in all brain regions whereas they remained constant in wild-type rats (Fig. 3g–l, o). In addition to these marked increases in numbers, the microglia surrounding the microvascular amyloid deposits showed a pronounced change in morphology. In 12-month-old wild-type rats, microglia are sparse and exhibit a resting, surveillance phenotype with long extended process (Fig. 3m). In contrast, the presence of microvascular amyloid promotes a distinct change in morphology to activated state with enlarged cell bodies and retracted processes that are closely engaged with the amyloid (Fig. 3n). These changes in microglia numbers and morphology were not evident in 1-month-old rTg-DI rats prior to microvascular amyloid deposition (Additional Fig. 2). At this young age, microglia were indistinguishable between wild-type and rTg-DI rats with both exhibiting a resting state with elaborate extended processes.

To investigate potentially different subtypes of microglia in rTg-DI rats, we performed double immunolabeling for Iba-1, a common resident microglial marker, and OX6 MHCII a marker that has been used to identify activated macrophagic microglia [22, 23]. In 3 and 12-month wild-type rats, the small number of microglia were labeled exclusively with Iba-1 and exhibited their resting state morphology (Additional Fig. 3). On the other hand, in the presence of microvascular amyloid at 3 months and more so at 12 months, a subset of microglia was labeled with both Iba-1 and OX6. However, there were few, if any, cells labeled solely with OX6. This suggests that a subpopulation of microglia with a potentially different activation state exists in rTg-DI rats, especially with advanced microvascular amyloid deposition. These series of data together show that robust increases in both astrocytes and microglia, presenting with perhaps different activation states, develop as CAA emerges and progresses in rTg-DI rats.

### Inflammatory marker expression in rTg-DI rat brain

Previous data from both human and transgenic mouse models of CAA have identified changes in expression of a number of inflammatory markers [1, 11, 12, 24–26]. Based on the inflammatory cell increases described above in rTg-DI rats, analysis of inflammatory marker expression could provide further insight into the pathogenesis of CAA in this model. Therefore, we next performed qRTPCR experiments to measure the expression of a series of inflammatory markers in the rTg-DI rat model of CAA at the early-stage disease onset (3 months) and at a stage of more advanced pathology (12 months).

**Fig. 4:**
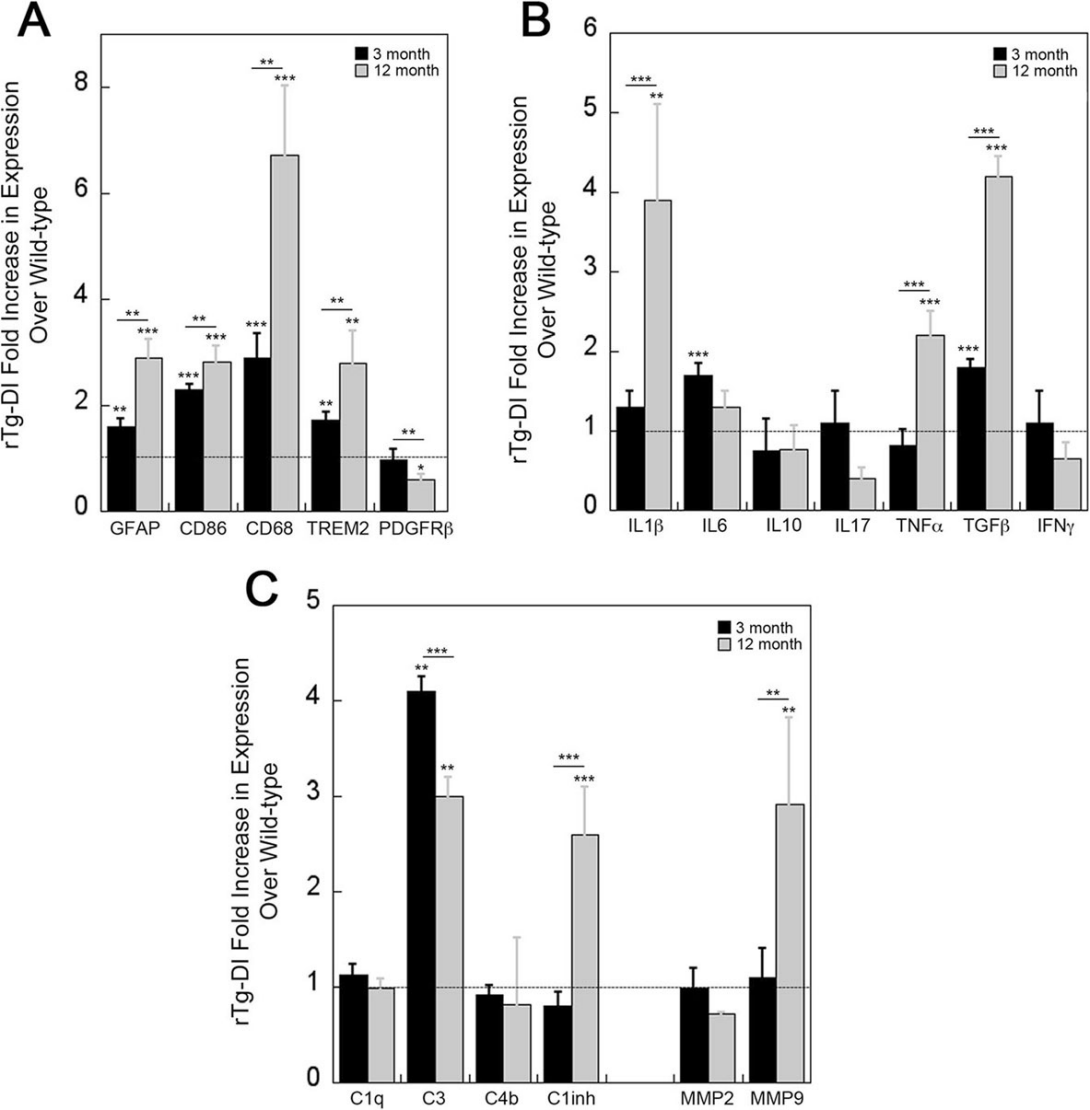
Inflammation-related gene expression in rTg-DI rat brains. Total RNA was extracted from 3 and 12 months old rTg-DI rats and wild-type rats followed by reverse transcription and real-time PCR analysis of marker gene expression was performed using β-actin as an internal control. Gene expressions in wild-type animals were normalized to 1 (dash line). Data presented are the means ± SD of *n* = 3–5 rats per group. *P < 0.05, **P < 0.01, ***P < 0.001

Since we observed significantly increased numbers of astrocytes and activated microglia in rTg-DI rat brains, as shown above in Figs. 2 and 3, we first focused on a set of cell type specific markers. Indeed, compared to wild-type rats, the rTg-DI rats exhibited significantly increased expression of GFAP at 3 months of age that further increased at 12 months of age (Fig. 4a). Similarly, we measured the activation markers cluster of differentiation 68 (CD68) and CD86 [27], as well as triggering receptor expressed on myeloid cells 2 (TREM2), and found all three genes were significantly elevated in rTg-DI rat brains at 3 months with the onset of CAA with further increases at 12 months with more advanced stage of microvascular amyloid (Fig. 4a). These findings of astrocyte and microglial marker gene expression is highly consistent with the increases in cell numbers and activation as shown above. Another cell marker measured was expression of platelet-derived growth factor receptor β (PDGFRβ), a marker for perivascular capillary pericytes which resides in the location vascular amyloid deposition in rTg-DI rats. With the emergence of CAA at 3 months in this model, there was no difference in PDGFRβ expression compared to wild-type rats (Fig. 4a). However, as vascular amyloid accumulation became more severe at 12 months there was a significant reduction in expression of this pericyte marker.

In previous studies, CD86 expression indicated classically activated, pro-inflammatory M1 microglia [28]. Therefore, we examined the mRNA levels of IL-1β, IL-6, and TNF-α in rTg-DI rat brains, which all participate in the polarization of M1 microglia [29]. Of these markers, only IL-6 showed a significant increase in expression at the onset of CAA, but this increase subsided as the rats presented with more advanced disease at 12 months (Fig. 4b). On the other hand, increased expression of IL-1β and TNF-α was not evident at early-stage disease but was significantly elevated at late-stage disease (Fig. 4b). Interestingly, expression of IL-17, which can upregulate microglial production of IL-1β and IL-6 [30], was not affected or appeared to be reduced in rTg-DI rats compared to wild-type rats. We also measured the expression of two important anti-inflammatory cytokines, IL-10 and transforming growth factor β (TGF-β). In this case, TGF-β showed a significant increase in expression at 3 months with a further increase at 12 months, while IL-10 showed somewhat lower expression in rTg-DI rats.

We next examined the expression of several complement cascade components and matrix-metalloproteinases (MMPs), which both can promote damage to cerebral blood vessels. For example, fibrillar Aβ deposits have been shown to increase expression and activate certain components of the complement cascade [31]. Interestingly, the expression levels of classical complement component 1q (C1q) and C4b did not show any significant difference between rTg-DI and wild-type rats at either stage of disease. On the other hand, the alternative pathway complement component 3 (C3) showed a robust increase in expression at onset of CAA accumulation that somewhat tapered at 12 months but was still highly significant. Expression of C1-inhibitor, which regulates complement activation, was significantly elevated in advanced disease, but not with the onset of microvascular CAA. Similarly, we found disparate expression of two key MMPs that can degrade vascular basement membranes and promote bleedings [32]. Whereas MMP9 expression was increased several-fold in rTg-DI rat brains at 12 months MMP2 was not. Taken together, these findings indicate that rTg-DI rats express a unique profile of inflammatory markers, complement components, and MMPs, some beginning at the onset of microvascular amyloid accumulation with more robust expression with increased pathology at 12 months, whereas others are only expressed at late stage of disease.

### Decreased perivascular pericytes in rTg-DI rats

**Fig. 5:**
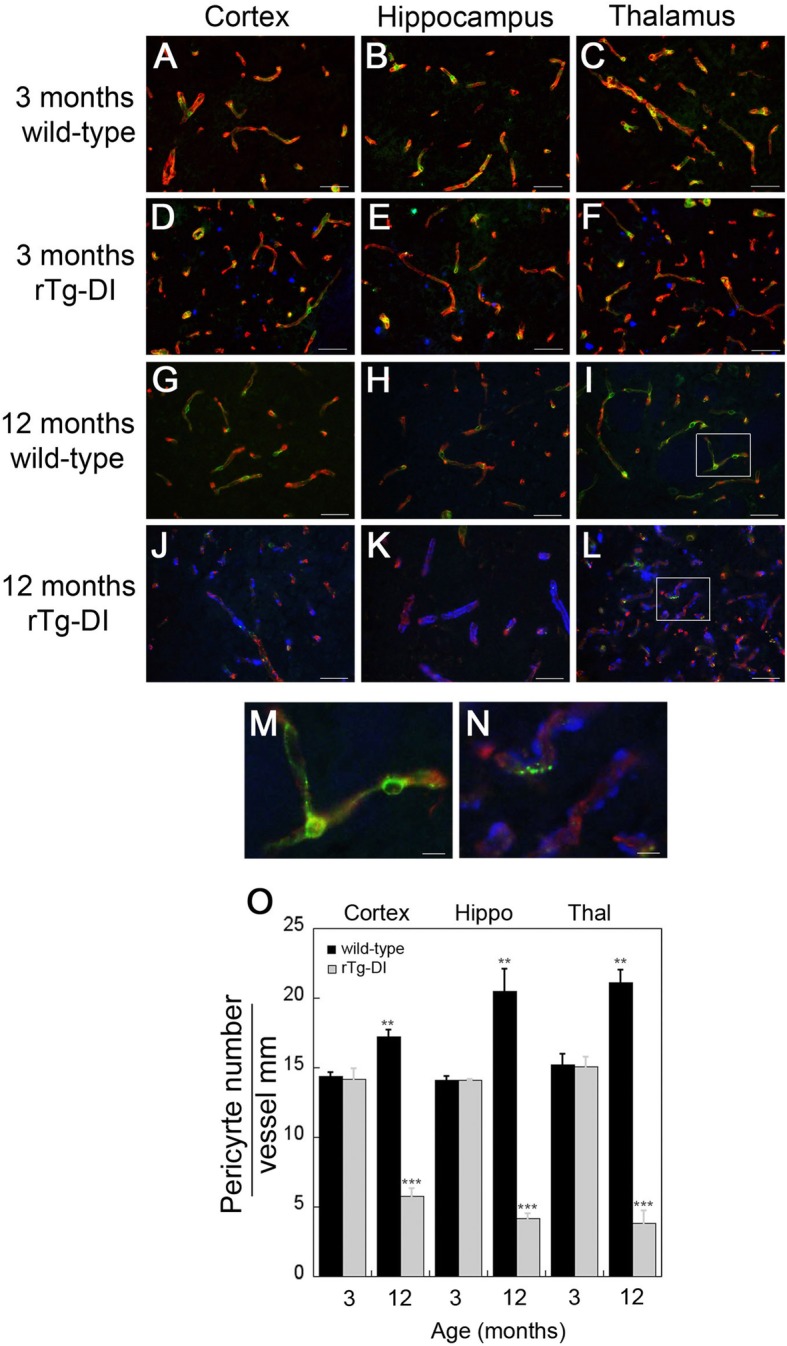
Loss of perivascular pericytes in rTg-DI rats. **a**–**l** Brain sections from 3-month-old wild-type (**a**–**c**) and rTg-DI (**d**–**f**) rats and 12-month wild-type (**g**–**i**) and rTg-DI (**j**–**l**) rats were labeled with Amylo-Glo to detect fibrillar amyloid (blue), rabbit polyclonal antibody to collagen IV to detect cerebral microvessels (red), and goat polyclonal antibody to PDGFRβ for pericytes (green). Scale bars = 50 μm. **m**, **n** Enlarged images of the highlighted regions of panels **i** and **l**, respectively. Scale bars = 10 μm. **o** Quantitation of pericyte numbers from wild-type rats (black bars) and rTg-DI rats (gray bars) in different brain regions at 3 and 12 months of age. Data shown are mean ± SD of *n* = 3–5 rats per group. Pericyte numbers on capillaries increased in wild-type rats in all brain regions as they aged from 3 to 12 months. **P < 0.002. In contrast, pericyte numbers markedly decreased in rTg-DI rats in all brain regions as they aged from 3 to 12 months. ***P < 0.001

Pericytes are vascular mural cells embedded in the basement membrane of blood microvessels, and uniquely positioned within the neurovascular unit between endothelial cells of capillaries [33, 34]. Previous studies in AβPP transgenic mice have shown that pericyte loss can impede soluble Aβ clearance and accelerate cerebral β-amyloidosis and CAA [35]. Further, our gene expression analysis in Fig. 4 showed that PDGFRβ, a marker for pericytes, is reduced at 12 months of age in rTg-DI rats. Therefore, we evaluated microvascular pericytes in rTg-DI rats at the different stages of the disease. In the wild-type rats, pericytes with extended processes were readily visualized along cerebral capillaries in all brain regions at 3 and 12 months of age (Fig. 5a–c, d–i, m). In fact, the number of pericytes along cerebral capillaries actually significantly increased from 3 to 12 months in wild-type rats (Fig. 5o). At 3 months of age, with the onset of CAA, the morphology and numbers of capillary pericytes in rTg-DI rats were indistinguishable from wild-type rats (Fig. 5d–f, o). In contrast, at 12 months with extensive capillary amyloid deposition, pericytes were markedly reduced, degenerative, and lacked long extended process (Fig. 5d–f, n). Quantitation of pericyte coverage in cerebral capillaries in the cortex, hippocampus, and thalamus of rTg-DI rats revealed a highly significant reduction (*p* < 0.001) in all brain regions. These findings are consistent with the decreased expression of PDGFRβ at 12 months of age in this model of microvascular CAA (Fig. 4a).

### Increased numbers of caspase 3-positive cells are associated with severe microvascular amyloid deposition in rTg-DI rats

**Fig. 6:**
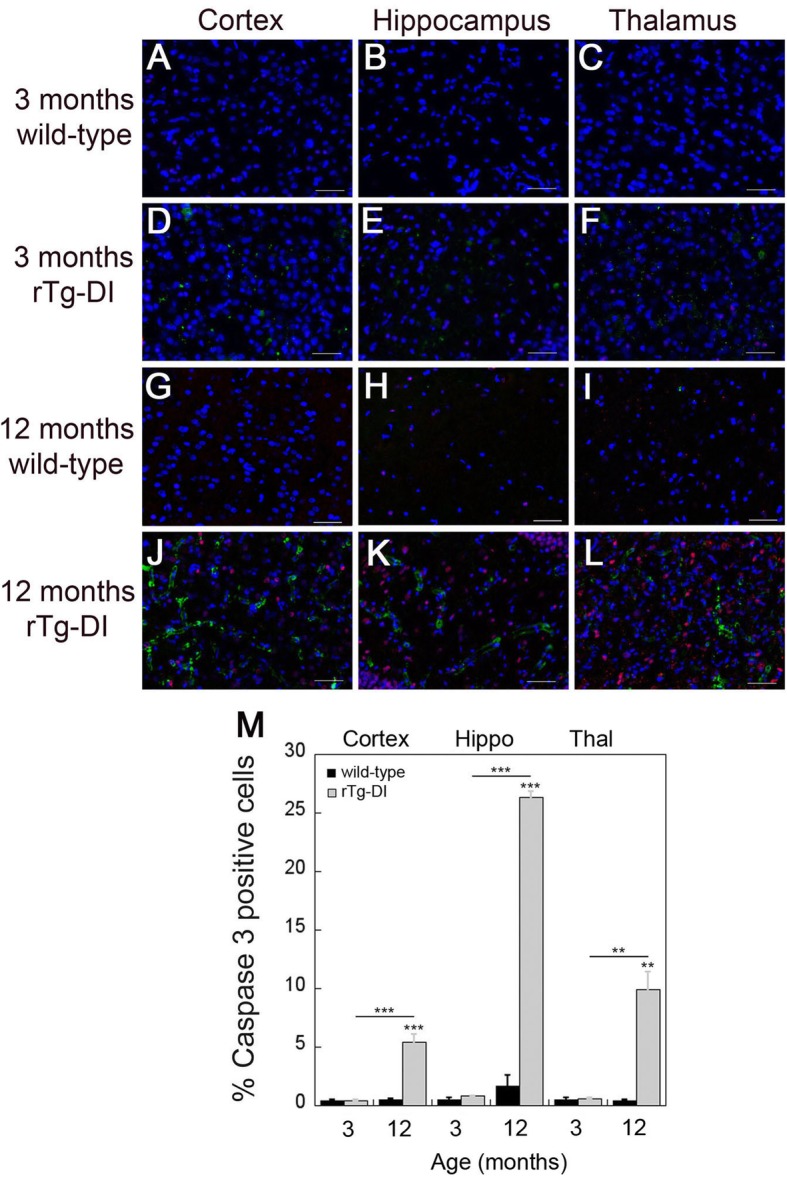
Progressive accumulation of microvascular amyloid leads to increased numbers of activated caspase-3 positive cells in rTg-DI rats. **a**–**f** Representative images from 3-month-old wild-type (**a**–**c**) and rTg-DI (**d**–**f**) rats and 12-month wild-type (**g**–**i**) and rTg-DI (**j**–**l**) rats. Brain sections were stained with DAPI (blue), immunolabeled with a rabbit polyclonal antibody to active caspase 3 to identify apoptotic cells (red) and with mouse monoclonal antibody 66.1 to identify cerebral microvascular amyloid (green). Scale bars = 50 μm. **g** Quantitation of activated caspase 3 positive cells in wild-type rats (black bars) and rTg-DI rats (gray bars) in the cortex, hippocampus, and thalamus. Data shown are mean ± SD of *n* = 5 rats per group. **P < 0.01, ***P < 0.001. In wild-type rats, very few activated caspase 3-positive cells were observed, but in rTg-DI rat brains, markedly elevated numbers of activated caspase 3-positive cells were seen surrounding microvascular amyloid in all brain regions

Our findings above show that the extensive cerebral microvascular amyloid accumulation at 12 months in rTg-DI rats is accompanied by robust neuroinflammation and loss of pericytes, recapitulating the pathological features of CAA type-1. In many human AβPP transgenic mouse models that develop cerebral amyloid deposits and promote cell stress, elevated caspase 3 activation was observed [36–38]. Multiple lines of evidence indicate that caspase 3 activation is both necessary and sufficient to trigger apoptosis [39, 40]. Since our above results clearly demonstrate that rTg-DI rats develop many signs of perivascular stress including strong neuroinflammation and loss of pericytes, we investigated if this also promoted caspase 3 activation in these brain regions. At 3 months of age, the wild-type and rTg-DI rats with the absence of microvascular CAA, exhibited little evidence for caspase 3 activation (Fig. 6a–f, m). However, at 12 months of age in the presence of extensive CAA type-1, there was a dramatic increase in caspase 3-positive cells in rTg-DI rats (Fig. 6 j–l). Quantitative measures showed that there were highly significant increases of caspase 3-labeled cells in the cortex, hippocampus, and thalamus, with abundant microvascular CAA (Fig. 6m). To identify what cell types express caspase 3 in the 12 months old rTg-DI rats, double labeling for caspase 3 and cell-specific markers was performed. Surprisingly, these studies showed that the majority of caspase 3-positive cells were found to be astrocytes, with little, if any, involvement of neurons or microglia (Additional Fig. 4).

### Disruption of axonal integrity in rTg-DI with advanced CAA pathology

**Fig. 7:**
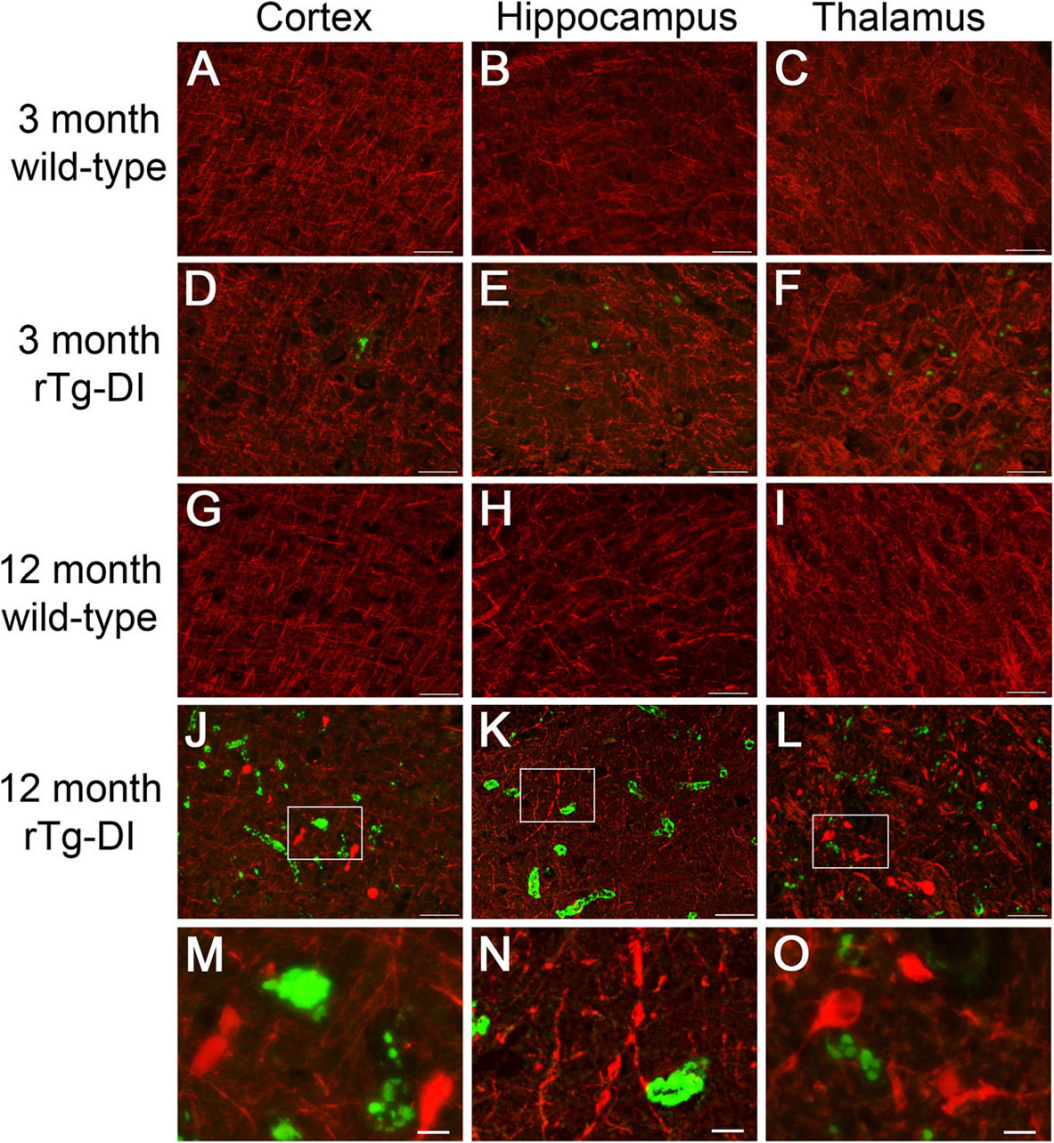
Disrupted axonal integrity in rTg-DI rats. **a**–**l** Brain sections from 3-month-old wild-type (**a**–**c**) and rTg-DI (**d**–**f**) rats and 12-month wild-type (**g**–**i**) and rTg-DI (**j**–**l**) rats were labeled with thioflavin S to identify fibrillar amyloid (green) and pan axonal neurofilament marker (red). Scale bars = 50 μm. **m**, **n**, **o** Enlarged images of the highlighted regions of panels **j**, **k**, and **l**, respectively. Scale bars = 10μm.

We next explored if advancing CAA pathology and associated neuroinflammation had effects on surrounding neurons. Although we found no evidence of overt neuronal loss in the brains of rTg-DI rats up to 12 months of age (not shown), we then focused on axonal integrity. At 3 months of age, when cerebral microvascular amyloid deposition first appears in rTg-DI rats, axonal morphology appeared very similar to that of wild-type rats (Fig. 7a–f). However, as rTg-DI rats aged to 12 months and presented with extensive microvascular amyloid there were noted changes to axonal integrity (Fig. 7j–o). For example, swollen and fragmented axons were evident. In addition, many neuronal cell bodies were now labeled with the axonal marker showing a striking redistribution of this reactivity. These findings suggest that there are indeed marked impacts of advancing CAA pathology on neuronal integrity, which likely contribute to behavioral deficits in rTg-DI rats [16].

## Discussion

CAA is a common cerebral small vessel disease of elderly people, a prominent feature of AD, and a cause of VCID. However, our understanding of the etiology and downstream pathological consequences of cerebral vascular amyloid accumulation are still limited resulting in a lack of effective therapeutic treatments. Therefore, valid and consistent preclinical animal models to study the pathogenesis of CAA are paramount. Most previous animal studies on CAA involved the use of various human AβPP transgenic mouse models that develop variable levels of CAA in the presence or absence of parenchymal amyloid pathology [41–44]. Recently, we reported the generation of a novel transgenic rat model (rTg-DI) that robustly develops capillary CAA type-1 [16]. We showed that rTg-DI rats express low amounts of human chimeric Dutch (E22Q)/Iowa (D23N) familial CAA mutant Aβ in the brain and develop early-onset and progressive microvascular CAA. As CAA progresses in rTg-DI rats, they develop consistent and numerous cerebral microbleeds that can readily be detected by magnetic resonance imaging. These findings suggest that rTg-DI rats have the potential to be a useful preclinical platform to study the pathogenesis of CAA type-1, to identify biomarkers for disease and to test therapeutic interventions. Indeed, we recently showed that decreasing levels of Aβ40 peptide in cerebrospinal fluid correlated with the progression of CAA in this model [22]. To better understand the utility of rTg-DI rats as a valid preclinical model, here we investigated the temporal development of several perivascular pathologies that are commonly observed with human CAA type-1. Our results show that with the progression of cerebral microvascular amyloid deposition, rTg-DI rats develop robust perivascular neuroinflammation, disruption and loss of capillary pericytes, increased numbers of caspase 3-positive cells, and disruption of axonal integrity. These findings indicate that rTg-DI rats faithfully recapitulate many of the pathological features of human CAA type-1 and provide new insight into the pathogenesis of this condition.

Neuroinflammation is recognized as a key component in the AD brain with marked increases and activation of glial cells surrounding parenchymal fibrillar amyloid plaques and expression of inflammatory mediators [45, 46]. Likewise, in human CAA type-1, where vascular amyloid protrudes into the perivascular brain parenchyma, activated astrocytes and microglia accumulate in response to the amyloid [12, 47]. Similarly, in rTg-DI rats, we found that corresponding with the increasing accumulation of cerebral microvascular amyloid there is a dramatic elevation in the numbers of astrocytes and activated microglia (Figs. 2 and 3). These glial responses emerged at the onset of microvascular amyloid deposition (≈ 3 months of age) and increased as the disease progressed to an advanced stage (12 months of age). Interestingly, in late-stage disease the astrocytes around microvascular amyloid deposits exhibited enlarged cell bodies and retracted process suggesting a degenerative phenotype. Indeed, subsequent experiments showed a significant increase in caspase 3-positive cells that were largely identified as astrocytes. This suggests a distinct cytotoxic effect of the microvascular amyloid to astrocytes in this model. Similar to the increase in astrocytes, there was a pronounced elevation in numbers of microglia in the vicinity of microvascular amyloid deposits. Morphologically, these microglia presented in an activated state with a noticeable retraction of the extensive processes commonly seen in resting microglia of wild-type rats. To better understand the state of these activated microglia, labeling for MHCII OX-6 was performed to identify macrophagic microglia [23, 48]. Clearly, there was a subset of microglia in rTg-DI rats that expressed both Iba-1 and OX-6 reactivity whereas many of the microglia only expressed Iba-1. This could suggest that dual-labeled microglia may derive from a different origin such as the periphery. However, recent studies suggest that microglia and macrophages can express similar or disparate markers depending on the environment where they exist (periphery vs brain) and the type of stimulus that leads to their activation [49]. Regarding this latter point, the activation stimulus here involves vascular amyloid and, in rTg-DI rats, involves familial Dutch/Iowa CAA mutant Aβ. Further specifying this pathogenic stimulus, we previously reported that in vascular amyloid, the Aβ peptide adopts a distinct anti-parallel fibril configuration [16, 50]. Thus, the source and activation state of microglia can be unique for distinct disease states in the CNS and requires further investigation in this model [51]. In any case, regardless of the source, there is clearly a large microglial response to the microvascular amyloid in rTg-DI rats.

As mentioned above, reactive gliosis is a common response to a variety of neurodegenerative diseases including AD, prion disease, Parkinson’s disease, and multiple sclerosis [52, 53]. In these diseases there exists a complex interaction between activated microglia and astrocytes mediated through pro- and anti-inflammatory cytokines/chemokines that can lead to neuronal dysfunction. Our findings show that in response to microvascular amyloid, the striking glial activation promoted a distinct pattern of inflammatory marker expression in rTg-DI rats (Fig. 4). For example, elevated expression of certain pro-inflammatory cell markers such as GFAP, CD86, CD68, and TREM2 were noted that reflect the elevated numbers of astrocytes and microglia. Each of these glial markers were elevated at the onset of CAA and further increased with progression of microvascular amyloid accumulation. Increased expression of TREM2 in rTg-DI rats is of interest since mutations in this gene are associated with an increased risk for AD [54, 55]. Our findings may suggest a role for TREM2 in the pathogenesis of CAA as well. Increases in pro-inflammatory IL-1β and TNF-α were noted, yet other pro-inflammatory markers such as IL-6, IL-17, and IFNγ did not increase with disease severity or were muted. On the other hand, TGF-β1, more associated with anti-inflammatory pathways, was also expressed at high levels but IL-10, another anti-inflammatory cytokine, was not. Interestingly, we also found increased expression of alternative pathway complement component C3 in rTg-DI rat brain, but not classical complement components C1q and C4, which have been shown to have a tighter relationship with Aβ plaque formation [31, 56]. C1-inhibitor, which regulates classical complement activation, was also increased in advanced disease. However, the lack of expression of classical complement components in rTg-DI suggest that elevated C1-inhibitor may rather be in response to disrupted vessel integrity and microbleeds that are present only in the later stages of disease. There are indications for C1-inhibitor playing a role in reducing cerebral thrombo-inflammation [57]. Along these lines, MMP9 also showed increased expression at advanced stage of disease, and this is likely associated with the microbleed phenotype associated with CAA. These results indicate that the pathogenesis of CAA type-1 in rTg-DI rats triggers neuroinflammation in the brain, but the gene expression pattern is unique, can be temporal, and differs from that of Aβ plaque-related neuroinflammation. Along these lines, we recently reported that cerebral vascular Aβ deposits have a unique, anti-parallel β-sheet fibril structure that is distinct from parenchymal plaque Aβ fibrils that possess a parallel β-sheet fibril structure [16, 50]. It is plausible that these distinct fibril structures promote distinct inflammatory signatures in the brain.

The spatial location of fibrillar amyloid deposits in CAA type-1 suggests direct interaction with pericytes, cells that regulate key neurovascular functions including blood-brain barrier formation and maintenance, clearance of toxic cellular byproducts, and regulating neuronal phenotype [58–60]. In rTg-DI rats, we found a dramatic reduction in the number of pericytes in the cortex, hippocampus, and thalamus at later stages of disease, consistent with the reduction in PDGFRβ expression. At the onset of CAA formation at 3 months, pericyte numbers and morphology were no different in rTg-DI rats compared with wild-type rats. In contrast, at 12 months there was considerable loss of extended processes along the length of the capillaries further underscoring the degenerative effects of accumulating amyloid on these cells. This is consistent with previous studies demonstrating that Aβ is toxic to pericytes in primary cell culture [61]. Accordingly, we suggest that there is likely a negative feedback loop between pericyte loss and CAA progression in rTg-DI rats where the loss of pericytes diminishes proper neurovascular function and clearance of soluble Aβ further promoting accumulation and deposition of Aβ on capillaries, which in turn amplifies amyloid-induced pericyte loss. Targeting this destructive interaction could have implications for developing strategies for early intervention in capillary CAA type-1.

We found a marked increase in caspase 3-positive cells indicating a marker for cell stress and pro-apoptotic processes. This increase in caspase 3-positive cells was observed in late-stage disease, but not early-stage disease suggesting that it results from the continuing accumulation of microvascular amyloid and the chronic neuroinflammation associated with it. It was interesting that these stressed caspase 3-positive cells were largely determined to be astrocytes further implicating the impact of vascular amyloid accumulation on this cell population. Although we have not observed overt neuronal loss in this model at 12 months of age, this could become apparent in older animals. However, at around 12 months of age rTg-DI rats generally become moribund thus preventing further aging. Nevertheless, we did find that at later stage disease, when caspase 3-positive astrocytes are abundant, there were some profound effects on axonal morphology with fragmentation, swelling, and redistribution of axonal labeling. This implicates the increasing microvascular amyloid burden, chronic inflammation, and perivascular disruption in compromising neuronal integrity that likely underlies behavioral deficits in rTg-DI rats leading to VCID in this model.

## Conclusions

The rTg-DI rat is a novel model of early-onset and progressive cerebral microvascular amyloid deposition that recapitulates many features of human CAA type-1. Our results show that there is a relationship between the onset and progressive accumulation of cerebral microvascular amyloid with the temporal development of neuroinflammation and perivascular cellular pathology. Advanced stages of microvascular amyloid and neuroinflammation in rTg-DI rats is associated with pronounced pericyte loss in capillaries, degeneration of astrocytes, and disruption of neuronal axonal integrity. These findings underscore the utility of rTg-DI rats to serve as a useful preclinical platform to develop biomarkers and to test therapeutic strategies to intervene in the onset and progression of microvascular CAA and its role in VCID.

## Supplementary information

**Additional file 1 : Figure S1.** Immunolabeling of astrocytes in 1 month old wild-type rats and rTg-DI rats prior to microvascular amyloid deposition. **A-F:** Brain sections from 1-month old wild-type (**A-C**) and rTg-DI (**D-F**) rats were labeled with Amylo-Glo to detect fibrillar amyloid (blue), rabbit polyclonal antibody to collagen IV to detect cerebral microvessels (red), and goat polyclonal antibody to GFAP to identify astrocytes (green). Scale bars = 10 μm. At this young age, in the absence of microvascular amyloid deposition astrocytes are morphologically indistinguishable between wild-type and rTg-DI rats.

**Additional file 2 : Figure S2.** Immunolabeling of microglia in 1 month old wild-type rats and rTg-DI rats prior to microvascular amyloid deposition. **A-F:** Brain sections from 1-month old wild-type (**A-C**) and rTg-DI (**D-F**) rats were labeled with Amylo-Glo to detect fibrillar amyloid (blue), rabbit polyclonal antibody to collagen IV to detect cerebral microvessels (red), and goat polyclonal antibody to Iba-1 to identify microglia (green). Scale bars = 10 μm. At this young age, in the absence of microvascular amyloid deposition microglia are morphologically indistinguishable between wild-type and rTg-DI rats with both exhibiting a resting phenotype.

**Additional file 3 : Figure S3.** Immunolabeling for macrophagic microglia in 3 and 12 month old wild-type rats and rTg-DI rats. **A-L:** Brain sections from 3-month old wild-type (**A-C**) and rTg-DI (**D-F**) rats and 12-month wild-type (**G-I**) and rTg-DI (**K-L**) rats were labeled with Amylo-Glo to detect fibrillar amyloid (blue), goat polyclonal antibody to Iba-1 as a marker for microglia (green) and mouse monoclonal antibody to OX6 as a marker for macrophages (red). Scale bars = 50 μm. In wild-type rats at both ages cells were solely labeled with Iba-1. In rTg-DI rats the majority of cells labeled solely with Iba-1 and a subset of cells were double labeled for the microglial marker Iba-1 and for the macrophagic microglial marker OX6. Few, if any, cells were labeled solely with OX6 antibody.

**Additional file 4 : Figure S4.** Double immunolabeling for caspase 3 and cell specific markers. **A-C:** Brain sections from 12 month old rTg-DI rats were immunolabeled for caspase 3 (red) and (**A**) NeuN to identify neurons (green), (**B**) GFAP to detect astrocytes (green) and (**C**) Iba-1 to identify microglia (green). Caspase 3 labeling most closely co-localized with astrocytes. Scale bars = 10 μm.

## Data Availability

The datasets used and/or analyzed during the current study are available from the corresponding author on reasonable request.

## Abbreviations

AD: Alzheimer’s disease
Aβ: Amyloid beta protein
AβPP: Aβ precursor protein
C1inh: Complement component 1 esterase inhibitor
C1q: Complement component 1q
C3: Complement component 3
C4b: Complement component 4b
CAA: Cerebral amyloid angiopathy
CD68: cluster of differentiation 68
CD86: cluster of differentiation 68
DAB: Diaminobenzidene
GFAP: Glial fibrillar acidic protein
Iba-1: Ionized calcium-binding adapter molecule 1
ICH: Intracerebral hemorrhage
IL-10: Interleukin-10
IL-17: Interleukin-17
IL-1β: Interleukin-1β
IL-6: Interleukin-6
MHCII: Major histocompatibility complex II
MMP2: Matrix metalloproteinase 2
MMP9: Matrix metalloproteinase 9
OCT: Optimal cutting temperature medium
PDGFRβ: Platelet-derived growth factor receptor β
PHF-1: Paired helical filament-1
rTg-DI: Transgenic rats that express Dutch/Iowa mutant Aβ in the brain
SD: Standard deviation
TGFβ1: Transforming growth factor β 1
TNF-α: Tumor necrosis factor alpha
TREM2: Triggering receptor expressed on myeloid cells 2
VCID: Vascular cognitive impairment and dementia
