# Supplementary figures and images for: Robust neuroinflammation and perivascular pathology in rTg-DI rats, a novel model of microvascular cerebral amyloid angiopathy
Source: J Neuroinflammation. 2020 Mar 4;17:78. doi: 10.1186/s12974-020-01755-y (PMC7055091; doi:10.1186/s12974-020-01755-y)

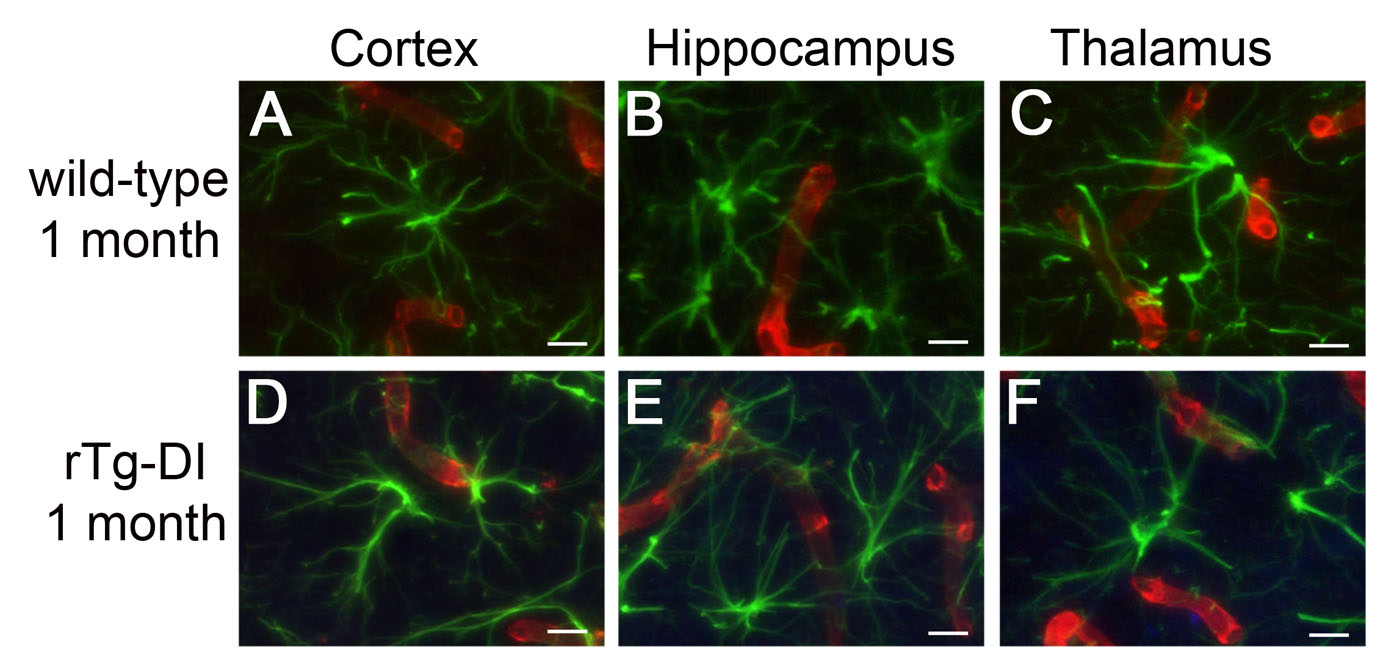

Supplement: Supplementary file 1 — Additional file 1 : Figure S1. Immunolabeling of astrocytes in 1 month old wild-type rats and rTg-DI rats prior to microvascular amyloid deposition. A-F: Brain sections from 1-month old wild-type (A-C) and rTg-DI (D-F) rats were labeled with Amylo-Glo to detect fibrillar amyloid (blue), rabbit polyclonal antibody to collagen IV to detect cerebral microvessels (red), and goat polyclonal antibody to GFAP to identify astrocytes (green). Scale bars = 10 μm. At this young age, in the absence of microvascular amyloid deposition astrocytes are morphologically indistinguishable between wild-type and rTg-DI rats. [file 12974_2020_1755_MOESM1_ESM.jpg]

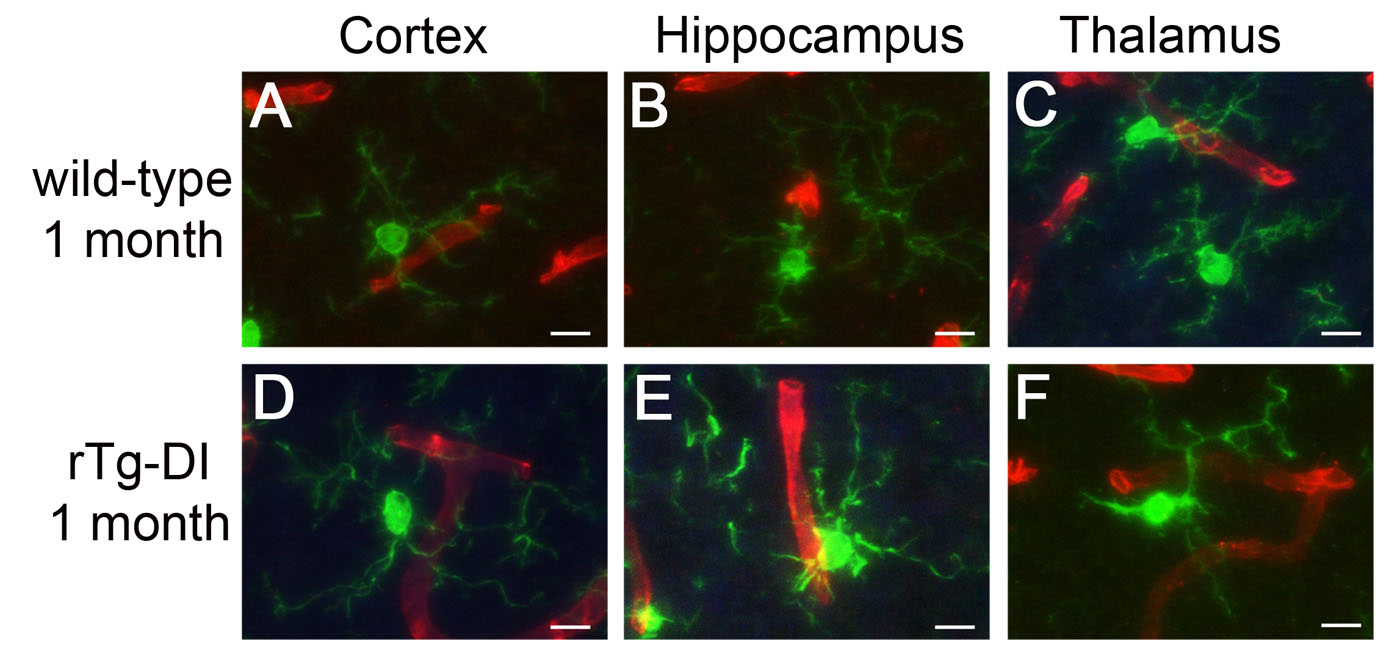

Supplement: Supplementary file 2 — Additional file 2 : Figure S2. Immunolabeling of microglia in 1 month old wild-type rats and rTg-DI rats prior to microvascular amyloid deposition. A-F: Brain sections from 1-month old wild-type (A-C) and rTg-DI (D-F) rats were labeled with Amylo-Glo to detect fibrillar amyloid (blue), rabbit polyclonal antibody to collagen IV to detect cerebral microvessels (red), and goat polyclonal antibody to Iba-1 to identify microglia (green). Scale bars = 10 μm. At this young age, in the absence of microvascular amyloid deposition microglia are morphologically indistinguishable between wild-type and rTg-DI rats with both exhibiting a resting phenotype. [file 12974_2020_1755_MOESM2_ESM.jpg]

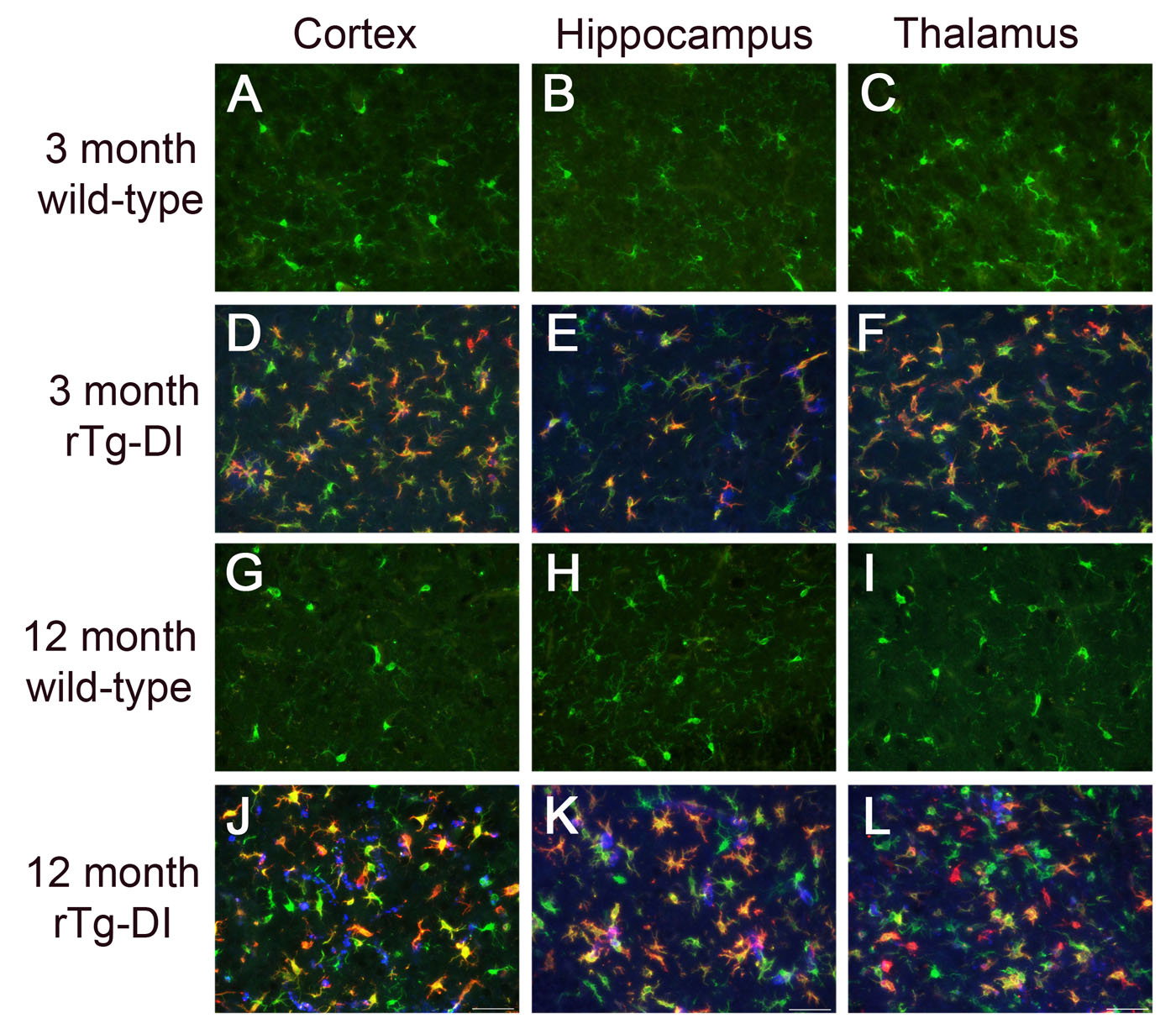

Supplement: Supplementary file 3 — Additional file 3 : Figure S3. Immunolabeling for macrophagic microglia in 3 and 12 month old wild-type rats and rTg-DI rats. A-L: Brain sections from 3-month old wild-type (A-C) and rTg-DI (D-F) rats and 12-month wild-type (G-I) and rTg-DI (K-L) rats were labeled with Amylo-Glo to detect fibrillar amyloid (blue), goat polyclonal antibody to Iba-1 as a marker for microglia (green) and mouse monoclonal antibody to OX6 as a marker for macrophages (red). Scale bars = 50 μm. In wild-type rats at both ages cells were solely labeled with Iba-1. In rTg-DI rats the majority of cells labeled solely with Iba-1 and a subset of cells were double labeled for the microglial marker Iba-1 and for the macrophagic microglial marker OX6. Few, if any, cells were labeled solely with OX6 antibody. [file 12974_2020_1755_MOESM3_ESM.jpg]

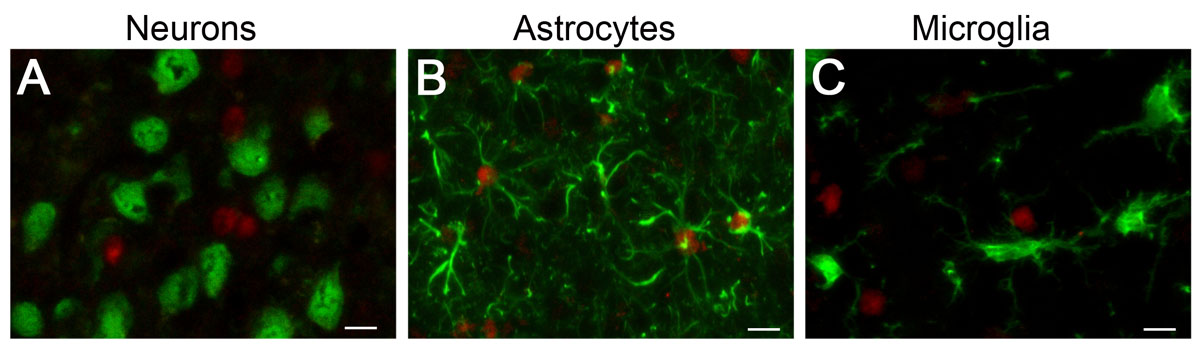

Supplement: Supplementary file 4 — Additional file 4 : Figure S4. Double immunolabeling for caspase 3 and cell specific markers. A-C: Brain sections from 12 month old rTg-DI rats were immunolabeled for caspase 3 (red) and (A) NeuN to identify neurons (green), (B) GFAP to detect astrocytes (green) and (C) Iba-1 to identify microglia (green). Caspase 3 labeling most closely co-localized with astrocytes. Scale bars = 10 μm. [file 12974_2020_1755_MOESM4_ESM.jpg]
